# Herbal and Dietary Supplement-Induced Liver Injury Compared With Conventional Drug-Induced Liver Injury: A Retrospective Cohort Study

**DOI:** 10.7759/cureus.106719

**Published:** 2026-04-09

**Authors:** Noor Albusta, Ahmed Ali, Ali Yusuf, Hussain Alrahma

**Affiliations:** 1 Internal Medicine, Beth Israel Lahey Health, Burlington, USA; 2 General Surgery, Salmaniya Medical Complex, Manama, BHR; 3 Medicine, Bahrain Government Hospitals, Manama, BHR; 4 Gastroenterology and Hepatology, Bahrain Government Hospitals, Manama, BHR

**Keywords:** acute liver failure, dietary and herbal supplements, drug-induced liver injury, epidemiology, hepatotoxicity, middle east

## Abstract

Introduction: Herbal and dietary supplements (HDS) are widely used globally and across the Middle East, yet their hepatotoxic potential remains underrecognized in this region. Comparable Bahrain-specific prevalence data are limited. This study aimed to quantify the proportion of drug-induced liver injury (DILI) attributable to HDS in a Bahraini cohort and to compare the clinical features and outcomes of HDS-related versus conventional medication-related liver injury, with severe liver injury as the primary outcome. We hypothesized that HDS-related DILI would be associated with more severe clinical outcomes than conventional medication-related DILI.

Methods: We conducted a retrospective cohort study of adults admitted with suspected DILI to Bahrain Government Hospital in Manama, Bahrain, between January 2018 and December 2024. Cases underwent structured causality assessment using the updated Roussel Uclaf Causality Assessment Method (RUCAM), and patients with RUCAM scores ≥6 for at least one implicated agent were included. Patients were classified into HDS-related or conventional medication-related liver injury groups according to the agent with the highest RUCAM score; cases with equal scores or unresolved competing exposures were excluded. Alternative causes of liver injury, including viral hepatitis and autoimmune liver disease, were excluded through routine clinical evaluation and available laboratory and imaging data. The primary outcome was severe liver injury, defined as coagulopathy, hepatic encephalopathy, or liver transplantation. Multivariable logistic regression was performed to identify independent predictors of severe liver injury.

Results: A total of 712 patients met the inclusion criteria, of whom 168 (23.6%) were attributed to HDS and 544 (76.4%) to conventional medications. Patients with HDS-related liver injury were younger (median age 34 vs. 52 years, p = 0.002) and more commonly female (63.2% vs. 41.1%, p < 0.001). Hepatocellular injury was more frequent in the HDS group (68.5% vs. 45.2%, p = 0.02). Severe liver injury occurred in 49/168 (29.2%) of HDS cases versus 79/544 (14.5%) of medication-related cases, corresponding to an absolute risk difference of 14.7% (95% CI 7.2-22.2). On multivariable analysis, HDS exposure remained independently associated with severe liver injury (adjusted OR 2.3, 95% CI 1.1-5.1, p = 0.03). Liver transplantation (7.7% vs. 1.7%, p = 0.08) and mortality (10.7% vs. 4.8%, p = 0.21) were numerically higher in the HDS group, although these differences did not reach statistical significance.

Conclusions: In this retrospective Bahraini cohort, HDS accounted for nearly one-quarter of RUCAM-confirmed DILI cases and was associated with a higher likelihood of severe liver injury than conventional medications. These findings support routine, structured inquiry about HDS exposure in patients presenting with liver injury and suggest that standardized medication and supplement history tools may improve early recognition. Larger prospective studies with product-level characterization are needed to better define the risks associated with HDS use in the region.

## Introduction

Herbal and dietary supplements (HDS) are increasingly used worldwide for a wide range of health-related purposes, with global consumption rising substantially over the past decade and market expenditures exceeding billions of dollars annually [1]. In Bahrain and across the Middle East, the use of herbal remedies, traditional medicines, and over-the-counter supplements is deeply embedded within cultural and social practices, often accompanied by the perception that these products are inherently safe due to their “natural” origin [2-7]. Population-based studies from Saudi Arabia report that 51.8-64% of adults consume dietary supplements, with particularly high usage among women, younger individuals, and those with higher education levels [2,3]. This prevalence further increased during the COVID-19 pandemic, when approximately 64% of individuals reported using supplements for immune enhancement [5]. However, this widespread perception of safety contrasts markedly with documented hepatotoxicity rates: studies from Western cohorts demonstrate that HDS account for 20% of all drug-induced liver injury (DILI) cases and are associated with higher rates of liver transplantation and mortality compared to conventional medications. Furthermore, regional data reveal that 49.9% of respondents use herbal medicines as first-line treatment when sick, and 42.1% do not consult physicians before use, creating substantial risk for unrecognized hepatotoxicity. Despite their widespread use, HDS are subject to less stringent regulatory oversight compared with conventional medications, and their safety profiles, particularly hepatotoxicity, remain incompletely characterized in many regions, including the Middle East [1,8].

DILI is a leading cause of acute liver injury and a major contributor to acute liver failure in developed countries, accounting for approximately 10% of acute hepatitis cases [9]. Over the past two decades, there has been a notable shift in the epidemiology of DILI, with an increasing proportion of cases attributed to HDS. Data from the Drug-Induced Liver Injury Network (DILIN) demonstrated that HDS-related liver injury increased from 7-9% of DILI cases in 2004-2007 to nearly 20% by 2010-2014 [9,10]. Furthermore, HDS-induced liver injury has been associated with more severe clinical outcomes, including higher rates of liver transplantation and mortality compared with conventional medication-induced injury [1,11,12]. In the United States, HDS accounted for over 20% of DILI-related acute liver failure cases in recent cohorts [13].

HDS-associated hepatotoxicity is often linked to multi-ingredient products marketed for weight loss, bodybuilding, or general well-being. These products frequently contain complex mixtures of vitamins, minerals, botanicals, and synthetic compounds, often with variable composition and uncertain quality [9]. Commonly implicated agents include anabolic steroids, green tea extract, Garcinia cambogia, and other proprietary blends, many of which have been associated with severe hepatocellular injury and acute liver failure [1,10]. The clinical phenotype is typically hepatocellular, which is associated with a higher risk of acute liver failure, transplantation, and mortality [9]. 

Despite growing evidence from Western cohorts, important regional data gaps remain regarding HDS-related hepatotoxicity in the Middle East. Specifically, there are limited Bahrain-specific data describing the proportion of DILI attributable to HDS, the clinical phenotype of injury, and comparative outcomes relative to conventional medication-related liver injury [2-7,14-16]. In addition, no comparable longitudinal regional data exist to determine whether the temporal rise in HDS-related DILI reported in U.S. cohorts is also occurring in Middle Eastern populations. Differences in supplement use patterns, healthcare access, referral pathways, and pharmacovigilance systems may limit direct comparison with Western cohorts.

Accordingly, this retrospective cohort study aimed to quantify the proportion of DILI cases attributable to HDS in a Bahraini hospital-based cohort and to compare the clinical presentation and outcomes of HDS-related versus conventional medication-related liver injury, with particular focus on severe liver injury. Given the observational design, the intent was to describe associations rather than infer causality. We hypothesized that HDS-related DILI would be associated with higher rates of severe liver injury, liver transplantation, and mortality compared to conventional medication-related DILI. 

## Materials and methods

Study design and setting

This retrospective cohort study was conducted at Bahrain Government Hospital, a tertiary care referral center in Bahrain, and included adult patients admitted between January 1, 2018, and December 31, 2024. Ethical approval was obtained from the institutional review board at Bahrain Government Hospital, and the requirement for informed consent was waived because of the retrospective design. Data were collected from electronic medical records in anonymized form prior to analysis.

Case identification and patient selection

Electronic medical records were reviewed to identify consecutive adult patients with suspected DILI. Liver injury was defined using standard biochemical thresholds consistent with published DILI criteria [9,17], including any of the following: alanine aminotransferase (ALT) or aspartate aminotransferase (AST) ≥5 times the upper limit of normal (ULN), alkaline phosphatase (ALP) ≥2 times ULN, or ALT/AST elevation accompanied by total bilirubin ≥2.5 mg/dL or international normalized ratio (INR) >1.5. 

Inclusion criteria were age ≥18 years, clinically suspected DILI within six months of exposure to a potentially hepatotoxic agent, and fulfillment of at least one biochemical criterion for liver injury, as well as Roussel Uclaf Causality Assessment Method (RUCAM) causality score ≥6 (probable or highly probable) for at least one implicated agent.

Exclusion criteria included acetaminophen toxicity, viral hepatitis, ischemic hepatitis, confirmed autoimmune hepatitis, primary biliary cholangitis, primary sclerosing cholangitis, chronic biliary obstruction, and prior liver transplantation, RUCAM score <6 for all potential causative agents, and cases with equal RUCAM scores for both HDS and conventional medications where a single predominant agent could not be determined. Alternative causes of liver injury were excluded on the basis of available clinical evaluation, serologic testing, imaging findings, and specialist documentation in the medical record. Confirmed autoimmune hepatitis was defined by the treating hepatology team based on the overall clinical assessment, including autoantibody profiles, immunoglobulin levels, compatible histology when available, and/or response to immunosuppressive therapy.

Causality assessment using RUCAM

All cases underwent systematic causality assessment using the RUCAM, a validated, structured instrument widely used by clinicians, the pharmaceutical industry, and regulatory agencies for DILI diagnosis [17]. RUCAM is a quantitative scoring system that evaluates seven key domains to determine the likelihood that a specific drug or HDS product caused the observed liver injury. The updated RUCAM (2016 version) generates scores ranging from -9 to +14, with separate scoring algorithms for hepatocellular injury (R >5) versus cholestatic or mixed injury (R ≤5) [17].

The seven RUCAM domains and their scoring ranges are as follows: 1) chronology (latency): time from drug initiation to liver injury onset and time from drug discontinuation to injury onset (+1 to +2 points); 2) dechallenge: improvement in liver biochemistries following drug discontinuation (-2 to +3 points for hepatocellular; 0 to +2 points for cholestatic/mixed); 3) competing causes: systematic exclusion of alternative diagnoses, including viral hepatitis (HAV, HBV, HCV, and HEV), autoimmune hepatitis, biliary obstruction, ischemic hepatitis, and other liver diseases (-3 to +2 points); 4) rechallenge: recurrence of liver injury upon re-exposure to the suspect agent (0 to +3 points); 5) known hepatotoxicity profile: published literature or product labeling documenting hepatotoxicity of the suspect agent (0 to +2 points); 6) risk factors: age >55 years, alcohol consumption, pregnancy (0 to +1 point); and 7) concomitant medications: assessment of other potentially hepatotoxic medications taken concurrently (-3 to 0 points).

RUCAM scores are interpreted according to five causality categories: highly probable (≥9 points), probable (6-8 points), possible (3-5 points), unlikely (1-2 points), and excluded (≤0 points).

For this study, RUCAM scoring was performed retrospectively by two independent trained investigators (N.A. and A.A.) using all available clinical data from electronic medical records, including detailed medication and supplement exposure history documented in admission notes, pharmacy records, and clinical assessments; temporal relationship between exposure initiation and liver injury onset; serial liver biochemistry values to assess dechallenge response; comprehensive serologic testing for viral hepatitis (anti-HAV IgM, HBsAg, anti-HBc IgM, anti-HCV, HCV RNA, anti-HEV IgM when available); autoimmune markers (ANA, ASMA, anti-LKM, and immunoglobulins); imaging studies (ultrasound, CT, and MRCP) to exclude biliary obstruction and vascular causes; liver biopsy results when available; documentation of rechallenge events (unintentional re-exposure); and published literature and FDA drug labeling information regarding hepatotoxicity of suspect agents.

Each case was scored independently by both investigators. Inter-rater reliability was assessed using the intraclass correlation coefficient (ICC). Discrepancies in RUCAM scores >2 points were resolved through consensus discussion, with involvement of a third investigator (H.A.) when necessary. For cases with multiple potential causative agents (HDS and/or conventional medications), separate RUCAM scores were calculated for each agent. Patients were classified according to the agent with the highest RUCAM score. Cases with equal RUCAM scores for HDS and conventional medications were excluded from analysis to minimize misclassification.

To ensure data quality and scoring consistency, all investigators completed formal training in RUCAM methodology using published guidelines and scoring examples prior to case adjudication. A standardized data extraction form was developed to systematically capture all RUCAM domains for each case.

Clinical data collection

Demographic and clinical data were extracted from electronic medical records, including age, sex, comorbidities, laboratory values at presentation, clinical manifestations, and outcomes. For HDS cases, product-specific information was extracted when available, including product name, intended use (weight loss, bodybuilding, general health), number of ingredients, duration of use, and dose. Because this was a retrospective study, the degree of underreporting of HDS exposure could not be measured, and some patients were unable to clearly recall product names, duration of use, or number of products taken. Chemical verification of HDS product contents was not available.

Pattern of liver injury

The pattern of liver injury was classified using the R ratio, calculated as (ALT/ULN) divided by (ALP/ULN). Hepatocellular injury was defined as R >5, mixed injury as 2 ≤ R ≤ 5, and cholestatic injury as R <2.

Outcomes

Primary outcomes included hospitalization, severe liver injury, liver transplantation, and mortality. Severe liver injury was defined as the presence of coagulopathy (INR ≥1.5), hepatic encephalopathy, or the need for liver transplantation. Outcomes were assessed during the index hospitalization and within 30 days of presentation.

Statistical analysis

Continuous variables were expressed as median with interquartile range, and categorical variables as frequency and percentage. Comparisons between groups were performed using the Mann-Whitney U test for continuous variables and chi-square or Fisher's exact test for categorical variables. Inter-rater reliability for RUCAM scoring was assessed using the intraclass correlation coefficient (ICC) with 95% confidence intervals.

Multivariable logistic regression was used to identify independent predictors of severe liver injury, adjusting for potential confounders including age, sex, pattern of injury, and baseline liver function tests. Variables for inclusion in the multivariable model were selected a priori based on clinical relevance and prior literature, including age, sex, HDS exposure, hepatocellular injury pattern, and baseline severity markers (ALT, total bilirubin, and INR). Prior to model construction, collinearity among liver function parameters was assessed using variance inflation factors (VIF); VIF values >5 were considered indicative of problematic collinearity. Model discrimination was evaluated using the C-statistic, and calibration was assessed using the Hosmer-Lemeshow goodness-of-fit test. Post-hoc power calculations were performed for non-significant outcomes using observed effect sizes and alpha=0.05.

Results were reported as odds ratios (OR) with 95% confidence intervals (CI). Given the exploratory nature of some comparisons, no formal adjustment for multiple testing was applied; however, p-values between 0.01 and 0.05 were interpreted with appropriate caution, and confidence intervals were provided to facilitate interpretation of effect magnitude. A p-value <0.05 was considered statistically significant. Statistical analyses were performed using IBM SPSS Statistics for Windows, version 28.0 (released 2021, IBM Corp., Armonk, NY).

## Results

**Figure 1:**
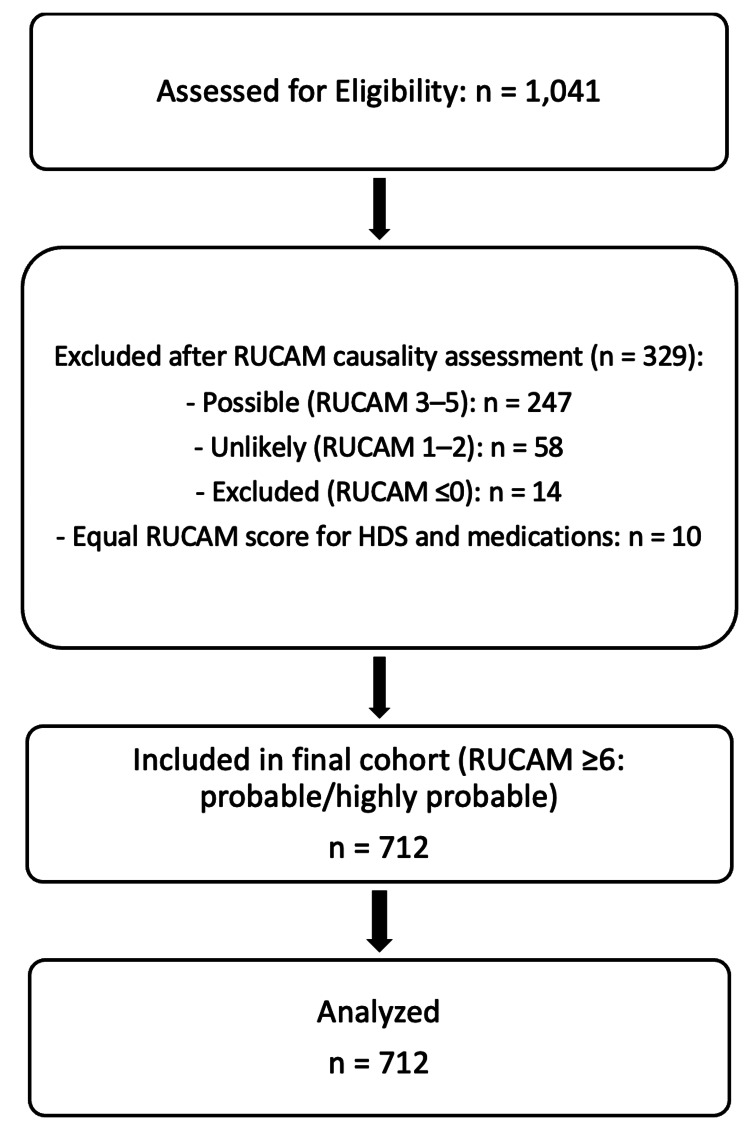
Flow diagram of patient selection

A total of 1,041 patients with suspected DILI were screened through electronic medical record review between January 2018 and December 2024. After applying the predefined inclusion and exclusion criteria, 712 patients met eligibility for the final cohort (Figure 1). After RUCAM causality assessment, 712 patients had RUCAM scores ≥6 (probable or highly probable) and met all other eligibility criteria for the final cohort (Figure 1). Among excluded cases, 247 had RUCAM scores 3-5 (possible), 58 had scores 1-2 (unlikely), 14 had scores ≤0 (excluded), and 10 had equal RUCAM scores for both HDS and conventional medications.

Inter-rater reliability for RUCAM scoring was excellent, with an intraclass correlation coefficient of 0.89 (95% CI 0.85-0.92). Among the 712 included cases, 142 (19.9%) were classified as highly probable (RUCAM ≥9) and 570 (80.1%) as probable (RUCAM 6-8).

Patients were subsequently classified according to the implicated exposure, with 168 cases (23.6%) attributed to HDS and 544 cases (76.4%) to conventional medications. Median RUCAM scores were 7 (interquartile range (IQR) 6-8) for HDS cases and 7 (IQR 6-9) for medication cases (p = 0.32), indicating comparable causality strength between groups. Among HDS cases, 28 (16.7%) had highly probable causality (RUCAM ≥9) compared to 114 (21.0%) of medication cases (p = 0.24).

Patients with HDS-related liver injury were significantly younger, with a median age of 34 years compared with 52 years in the medication group (p = 0.002), and were more likely to be female (63.2% vs. 41.1%, p < 0.001). Patients with HDS-related liver injury demonstrated higher transaminase levels at presentation, with a median ALT of 820 U/L compared with 520 U/L in the medication group (p = 0.01) and AST of 710 U/L versus 480 U/L (p = 0.02). By contrast, cholestatic markers were more elevated in the medication group, with a higher median ALP (310 U/L vs. 180 U/L, p = 0.01). Total bilirubin and INR were comparable between groups, with no statistically significant differences (bilirubin 6.5 vs. 5.2 mg/dL, p = 0.09; INR 1.4 vs. 1.3, p = 0.15) (Table 1). Although these differences did not reach statistical significance, the absolute values suggest clinically meaningful differences in baseline severity. Post-hoc power analysis indicated that the study had 45% power to detect the observed difference in total bilirubin and 38% power for INR, suggesting these comparisons were underpowered to detect potentially clinically relevant differences.

**Table 1:** Baseline characteristics and laboratory findings of patients with HDS versus medication-induced liver injury Continuous variables are presented as median (IQR) and were compared using the Mann-Whitney U test; standardized Z values are reported. Categorical variables are presented as n (%) and were compared using the chi-square test (χ² ). HDS: herbal and dietary supplements, RUCAM: Roussel Uclaf Causality Assessment Method, ALT: alanine transaminase, AST: aspartate transaminase, ALP: alkaline phosphatase, INR: international normalized ratio, IQR: interquartile range

Variable	HDS (n = 168)	Medications (n = 544)	Test statistic	p-value
RUCAM score (median, IQR)	7 (6–8)	7 (6–9)	Z = -1.0	0.32
RUCAM ≥9 (highly probable), n (%)	28 (16.7%)	114 (21.0%)	χ² = 1.4	0.24
Age, years (median, IQR)	34 (26–45)	52 (40–64)	Z = -3.1	0.002
Female sex, n (%)	106 (63.2%)	224 (41.1%)	χ² = 24.8	<0.001
Laboratory values at presentation (median, IQR)				
ALT (U/L)	820 (450–1350)	520 (280–940)	Z = -2.6	0.01
AST (U/L)	710 (380–1200)	480 (260–880)	Z = -2.3	0.02
ALP (U/L)	180 (110–320)	310 (180–540)	Z = -2.6	0.01
Total bilirubin (mg/dL)	6.5 (3.2–12.0)	5.2 (2.8–9.5)	Z = -1.7	0.09
INR	1.4 (1.1–1.9)	1.3 (1.1–1.7)	Z = -1.4	0.15

Among the 168 HDS cases, specific product information was available for 142 cases (84.5%). The most commonly implicated categories were: weight loss supplements (n = 48, 33.8%), including products containing green tea extract, *Garcinia cambogia*, and multi-ingredient thermogenic formulas; bodybuilding and muscle-building supplements (n = 31, 21.8%), including protein powders with added botanicals and anabolic steroid-containing products; traditional herbal remedies (n = 28, 19.7%), including Arabic and Ayurvedic preparations; turmeric/curcumin supplements (n = 15, 10.6%); and other single or multi-ingredient supplements (n = 20, 14.1%). In 26 cases (15.5%), patients reported HDS use but could not recall specific product names or brands.

Hepatocellular injury was the predominant pattern in the HDS group, occurring in 115 (68.5%) of cases compared with 246 (45.2%) in the medication group (p = 0.02), whereas cholestatic injury was more frequently observed among medication-related cases. Clinical outcomes are summarized in Table 2. Hospitalization occurred in 119 (70.8%) HDS cases compared with 320 (58.8%) medication-related cases (p = 0.18). Severe liver injury was significantly more common in the HDS group (49 (29.2%) vs. 79 (14.5%), p = 0.04); the absolute risk difference was 14.7% (95% CI 7.2-22.2%), indicating that approximately one in seven additional patients with HDS-related DILI experienced severe liver injury compared to those with conventional medication-related DILI. Liver transplantation was required in 13 (7.7%) HDS cases compared with nine (1.7%) in the medication group (p = 0.08). Mortality was higher in the HDS group (18 (10.7%) vs. 26 (4.8%)), although this difference did not reach statistical significance (p = 0.21). Post-hoc power analysis indicated 68% power to detect the observed difference in transplantation rates and 52% power for mortality, suggesting these outcomes were underpowered.

**Table 2:** Clinical outcomes of HDS vs. medication-induced liver injury Categorical variables are presented as n (%) and were compared using the chi-square test; Fisher’s exact test was used where appropriate for small cell counts. HDS: herbal and dietary supplement

Outcome	HDS (n = 168)	Medications (n = 544)	Test statistic	p-value
Hospitalization	119 (70.8%)	320 (58.8%)	χ² = 1.80	0.18
Severe liver injury	49 (29.2%)	79 (14.5%)	χ² = 4.22	0.04
Liver transplantation	13 (7.7%)	9 (1.7%)	Fisher’s exact	0.08
Mortality	18 (10.7%)	26 (4.8%)	χ² = 1.56	0.21

On multivariable logistic regression analysis, HDS exposure remained independently associated with severe liver injury (adjusted OR 2.3, 95% CI 1.1-5.1, p = 0.03). In addition, a hepatocellular pattern of injury was a strong independent predictor (adjusted OR 2.8, 95% CI 1.7-4.6, p < 0.001). Higher baseline liver injury markers, including ALT (per 100 U/L increase: OR 1.05, 95% CI 1.01-1.09, p = 0.01), total bilirubin (per mg/dL increase: OR 1.08, 95% CI 1.02-1.14, p = 0.008), and INR (per unit increase: OR 2.1, 95% CI 1.4-3.2, p < 0.001), were also independently associated with severe liver injury. Age and female sex were not significantly associated with the outcome after adjustment (p = 0.18 and p = 0.07, respectively). The final multivariable model demonstrated good discrimination (C-statistic = 0.78, 95% CI 0.74-0.82) and acceptable calibration (Hosmer-Lemeshow χ² = 11.2, p = 0.19). All variance inflation factors were <3.0, indicating acceptable collinearity among predictor variables. These findings are summarized in Table 3.

**Table 3:** Multivariable logistic regression for severe liver injury HDS: herbal and dietary supplement, ALT: alanine aminotransferase, INR: international normalized ratio

Variable	β (coefficient)	Adjusted OR (95% CI)	p-value
HDS exposure (vs medications)	0.83	2.3 (1.1–5.1)	0.03
Age (per year increase)	-0.02	0.98 (0.96–1.01)	0.18
Female sex	0.41	1.51 (0.97–2.35)	0.07
Hepatocellular pattern (vs others)	1.03	2.8 (1.7–4.6)	<0.001
ALT (per 100 U/L increase)	0.05	1.05 (1.01–1.09)	0.01
Total bilirubin (per mg/dL increase)	0.08	1.08 (1.02–1.14)	0.008
INR (per unit increase)	0.74	2.1 (1.4–3.2)	<0.001

## Discussion

In this retrospective study from Bahrain, HDS accounted for nearly one-quarter (23.6%) of all RUCAM-confirmed DILI cases, highlighting their substantial contribution to liver injury in this regional cohort. This proportion is consistent with data from the United States and other Western countries, where HDS comprises approximately 20% of DILI cases, suggesting that HDS-associated hepatotoxicity is not confined to Western settings and may represent an underrecognized problem across diverse healthcare systems [1,10,11,12,18].

Demographic and clinical characteristics

Patients with HDS-induced liver injury in our cohort were significantly younger (median age 34 years) and more commonly female (63.2%), consistent with patterns observed in Western cohorts [11-12,19]. A study from the Latin American DILI Network (LATINDILI) reported a mean age of 45 years with 66% female predominance among HDS cases, while the Spanish DILI Registry found 63% female representation [11,18]. The younger age in our Bahraini cohort may reflect regional patterns of HDS use, particularly among younger women seeking weight loss or cosmetic benefits, as documented in Saudi Arabian studies showing high HDS use among younger, educated females [2-3]. The female predominance may be explained by higher rates of HDS consumption among women for weight management, general health promotion, and cosmetic purposes [1-3].

Pattern of injury and severity

The predominance of hepatocellular injury in the HDS group (68.4%) is clinically significant and consistent with international data. The American College of Gastroenterology guidelines note that most HDS cause hepatocellular-type liver injury (R > 5), with the exception of bodybuilding products containing anabolic steroids, which typically cause cholestatic injury [18]. This hepatocellular pattern is particularly concerning because it has been associated with a higher risk of acute liver failure and the need for transplantation [9]. The AASLD Practice Guidance emphasizes that the clinical phenotype of liver injury in most cases associated with HDS is acute hepatocellular hepatitis, which is often severe, with a high rate of fulminant hepatic failure [9].

Importantly, HDS-related liver injury in our cohort was associated with a significantly higher proportion of severe liver injury (29.2% vs. 14.5%, p = 0.04), corresponding to an absolute risk difference of 14.7% (95% CI 7.2-22.2). In multivariable analysis, HDS exposure remained associated with severe liver injury (adjusted OR 2.3, 95% CI 1.1-5.1) after controlling for age, sex, injury pattern, and baseline severity markers. However, given the retrospective observational design and the possibility of residual confounding from unmeasured variables such as dose, duration of exposure, time to presentation, and product-specific toxicity, these findings should be interpreted as associations rather than causal effects. This finding aligns with multiple international studies demonstrating that patients with HDS hepatotoxicity leading to liver failure are more likely to die or undergo transplantation compared to patients with conventional drug hepatotoxicity [1,11-12]. The LATINDILI Network reported that HDS hepatotoxicity had the highest frequency of fatal/liver transplantation outcomes (21%) compared to conventional medications (12%) and anabolic steroids (13%) [11]. Similarly, the U.S. DILIN found that liver injury from non-bodybuilding HDS led to death or transplantation in 13% of cases versus only 3% for conventional medications [12].

Mechanisms of hepatotoxicity

The mechanisms underlying HDS hepatotoxicity are not fully understood and likely involve multiple pathways, including idiosyncratic reactions, direct toxicity, mitochondrial injury, and immune-mediated damage [1,9,10]. Among the HDS cases in our cohort with available product-level data, the most commonly implicated categories included weight-loss supplements, bodybuilding products, traditional herbal remedies, and turmeric/curcumin preparations. This profile is broadly consistent with prior literature linking multi-ingredient supplements and hepatocellular injury to severe outcomes [1,9,10,18]. However, because chemical verification of products was not available, specific ingredient-level attribution remains uncertain.

Regional context and cultural factors

In Bahrain and the broader Gulf region, the widespread use of traditional remedies and over-the-counter supplements without medical supervision may further contribute to risk [2-7]. Studies from Saudi Arabia demonstrate that 49.9% of respondents used herbal medicines as a first choice when sick, and 42.1% did not consult doctors before taking herbal medicines [6]. This pattern of unsupervised use, combined with the perception that herbal products are inherently safe, creates a perfect storm for unrecognized hepatotoxicity [6-7]. Furthermore, 73.3% of individuals with chronic conditions in Saudi Arabia practiced self-medication, including with HDS products, potentially increasing the risk of drug-HDS interactions and compounding liver injury [7].

Delayed recognition and outcomes

The higher mortality and transplantation rates observed in HDS cases, although not statistically significant in our study, align with the hypothesis that delayed recognition of the product as the cause of liver injury or reluctance of HDS consumers to seek medical care contributes to worse outcomes [1]. Patients may not volunteer information about HDS use, viewing these products as "natural" and therefore irrelevant to their medical condition [18]. The ACG Clinical Guideline emphasizes that clinicians must specifically query patients about their use of HDS, realizing that many will not be forthcoming with this history [18]. In addition, the latency period for HDS-induced liver injury may be quite prolonged in some instances, further complicating diagnosis [18].

Regulatory challenges

The regulatory framework relevant to HDS safety in Bahrain and the broader Middle East remains incompletely characterized in the published literature. More broadly, international experience suggests that variable oversight, inconsistent labeling standards, and limited product verification can contribute to underrecognized hepatotoxicity [8,14-16,20]. These challenges are especially important in settings where patients may use over-the-counter or traditional products without medical supervision.

Public health implications

Our findings have several practical implications. First, clinicians evaluating unexplained liver injury should use structured medication-history tools that specifically ask about non-prescribed supplements, traditional remedies, weight-loss products, and bodybuilding agents. Second, standardized screening protocols for HDS exposure may improve recognition in acute presentations. Third, patient education is needed to address the persistent misconception that “natural” products are inherently safe. Finally, region-specific pharmacovigilance and DILI registries may help clarify the burden, products involved, and outcomes of HDS-related hepatotoxicity in Middle Eastern populations [11-12,19,21].

Limitations

This study has several limitations. First, the retrospective single-center design introduces the possibility of selection bias, referral bias, and incomplete data capture. Second, although all included cases underwent structured causality assessment using RUCAM, retrospective scoring depended on the completeness and accuracy of documentation in the medical record, and some degree of exposure misclassification remains possible, particularly in patients with multiple potential hepatotoxic agents. Third, HDS exposure may have been underreported, and the degree of underreporting could not be measured; in addition, patients often could not clearly recall product names, duration of use, dose, or the number of products taken. Fourth, product-specific characterization was incomplete, and chemical verification of implicated HDS products was not available, limiting mechanistic interpretation. Fifth, uncommon outcomes such as liver transplantation and mortality were based on relatively small event counts, reducing the power to detect significant differences despite clinically relevant absolute differences. Finally, although model diagnostics were assessed and collinearity appeared acceptable, residual confounding cannot be excluded in an observational study of this type.

Future directions

Future research should focus on prospective studies with systematic collection of HDS exposure data, including product samples for chemical analysis. Genetic studies examining HLA associations with HDS hepatotoxicity in Middle Eastern populations would be valuable, given the known association of HLA-B35:01 with green tea extract hepatotoxicity in other populations [1]. Collaboration with regional poison control centers and establishment of a Middle Eastern DILI registry would enhance surveillance and characterization of HDS-induced liver injury. Finally, interventional studies examining the effectiveness of educational campaigns and regulatory interventions in reducing HDS hepatotoxicity are needed.

## Conclusions

In this retrospective Bahraini cohort, HDS accounted for a substantial proportion of DILI cases and were associated with a higher likelihood of severe liver injury than conventional medications. These findings support routine inquiry about HDS exposure in patients presenting with unexplained liver injury. Given the observational design and potential for exposure misclassification and residual confounding, larger prospective studies with standardized causality assessment and product-level characterization are needed to better define the burden and risks of HDS-related hepatotoxicity in the region.
